# Introducing a Chatbot to Support Victim-Survivors of Domestic Abuse: Victim-Survivor Perspectives

**DOI:** 10.1177/10778012251363583

**Published:** 2025-08-04

**Authors:** Nancy Lombard, Kate Butterby, Hanna Mielismäki, Rocío Vicente-García, Vanesa Pérez-Martínez

**Affiliations:** 1 70447 Glasgow Caledonian University, Glasgow, UK; 2 7840 Tampere University, Tampere, Finland; 3 16718 University of Alicante, San Vicente del Raspeig, Spain

**Keywords:** chatbot, digital technology, domestic abuse, help-seeking

## Abstract

The use of mobile-based technology to support victim-survivors who are subjected to domestic violence and abuse (DVA) is becoming more prevalent. This article explores the challenges that women who are seeking help for DVA may experience, examining whether a chatbot can offer positive solutions alongside highlighting some of the apprehensions victim-survivors have about its creation and implementation including safety and privacy concerns. We conclude that the chatbot has the potential to be an innovative solution but could also be detrimental for survivors if used as an alternative to investment in preventative, transformative innovations focusing on eliminating DVA.

## Introduction

The use of mobile-based technology to support victim-survivors who are subjected to domestic violence and abuse (DVA) is becoming more prevalent, with 24/7 availability and ease of use reported as positives. Although some countries are using supportive technology to increase knowledge about DVA or to facilitate reporting of DVA (e.g., hotlines and chatbots),^1^ no studies have been identified that explore the opinions of victim-survivors about the development and potential uses of a chatbot.

This article discusses the development of a chatbot to support women who are subjected to DVA as part of a wider project, Innovative Solutions to Eliminate Domestic Abuse (ISEDA), which consists of 15 partners from 9 European Countries (only 7 were included in this work package). The wider project aims to use multi-sector expertise via modern technological tools and practices in order to tackle and eliminate DVA. One such tool is the chatbot for victim-survivors who are subjected to DVA, discussed in this paper. Chatbots are defined as: “A computer program designed to simulate conversation with a human user, usually over the internet” (Oxford University Press, n.d.).

We begin this article by exploring general literature relating to DVA and technology, outlining the possibilities and concerns for women, before focusing more specifically on our study.

## Literature Review

### Domestic Abuse and Help-Seeking

Women seeking help do so from a range of sources and avenues. Not all victim-survivors want to involve the police, in part due to the complexities and nuances of reporting a person you are (or have been) intimately connected to (see Belknap & Hartman, 2000; Hester, 2011; Hester & Westmarland, 2005; Lombard et al., 2023) alongside fears of possible repercussions (Wolf et al., 2003). Research also demonstrates that women wait until they feel safe before speaking out (Lombard et al., 2023). In Finland, for example, DVA is highly unreported (Husso et al., 2021; Niklander et al., 2019; Siltala et al., 2023; Virkki et al., 2015). Up to 44% of women who have experienced violence do not tell anyone about it and only a tenth of women report it to the police (Statistics Finland, 2023).

Help-seeking is not a single act, but rather a continuous and complex process (Anderson & Saunders, 2003). Research tells us that when they have been subjected to violence and abuse, women first disclose to informal sources, with friends being the most common (Ahrens et al., 2009; Donovan et al., 2014; Fehler-Cabral & Campbell, 2013; Filipas & Ullman, 2001). Friends are particularly important to provide emotional support to victim-survivors (Sultana et al., 2023) and can be easier to approach for help than formal sources (Gregory, 2014). However, Evans and Feder (2015) found that these informal disclosures were only likely to lead to more formal support measures if their friends and family had knowledge or experience of DVA. They also found that women generally sought out formal help when they had reached a crisis point and did so from either the police or more general housing support services.

#### Barriers to seeking help

Barriers to help-seeking for victim-survivors include emotional ones (fear, embarrassment/shame, and self-blame), physical constraints (partner's physical presence, controlling behavior, and manipulation of professionals), and organizational (the appropriateness of the setting and time for disclosure). All of these negative experiences may hinder reporting (Husso et al., 2021; Krug et al., 2002; Lombard & Proctor, 2024; Piippo et al., 2021) and more so when there are a range of intersectional disadvantages to compound this.

According to Lombard and Scott (2013), older women experiencing abuse are more likely to experience self-blame, powerlessness, and hopelessness. Further barriers to reporting include the obligation to protect family, and the desire to keep such abuse secret from others (Beaulaurier et al., 2005). Ethnically minoritized and immigrant women face additional barriers because of institutional racism, immigration laws, culture and religion, issues of cultural competence, and lack of diversity within frontline services (Hulley et al., 2023). Additional problems for ethnically minoritized women can be language difficulties and religious practices (see Heron et al., 2022).

For lesbian, gay, bisexual and / or transgender+ (LGB and / or T+) victim-survivors, barriers to seeking help include not recognizing themselves and their experiences in the “public story”^2^ of DVA, their perpetrator's experiential power (telling the victim-survivor how LGB and/or T+ relationships are, which can essentially normalize DVA) and fear that formal services would not understand their identities or believe them (Donovan & Hester, 2014).

### Domestic Abuse and Current Technology Solutions

Research has found that technology can be helpful in supporting victim-survivors who have been subjected to DVA. Technology is more readily available than support services, meaning it can be accessed in accordance with victim-survivors’ schedules; a particular positive for those who are more isolated and who may find it difficult to access in-person support services (Emezue et al., 2022; Tarzia et al., 2017). It can also feel more private, less judgmental, and less intimidating than speaking to someone face-to-face (Glass et al., 2017; Lucas et al., 2014; Storer et al., 2022). There is also suggestion that it is easier for a victim-survivor to open up to technology when compared to a person (Lucas et al., 2014; Pickard et al., 2016). Young people may particularly benefit from technological tools due to their familiarity with using technology in general (Park & Lee, 2021; Storer et al., 2022; Tarzia et al., 2017) and recent research has found that young people like the idea of using a chatbot to tackle online abuse (Piccolo & Alani, 2020).

Negatives of technology for victim-survivors who are subjected to DVA have also been explored through research. Brignone and Edleson (2019) carried out an evaluation of existing DVA applications [apps] and found that many were outdated and contained broken links; a risk when developing and maintaining technology. Other findings suggest that as technology does not have the capacity to display human emotion and characteristics, such as empathy and reading body language, and should be used to direct people to human-led support services rather than replacing them (Domingo-Cabarrubias et al., 2023; Tarzia et al., 2017; Xu et al., 2021). Some victim-survivors may not have access to technology due to socio-economic constraints whereas other minoritized victim-survivors such as those who are neurodiverse, older, or deaf, or those who struggle with literacy may not be able to access some types of technology. Research suggests that many DVA apps are in the English language, meaning they are not accessible to victim-survivors who do not speak or understand English (Domingo-Cabarrubias et al., 2023). The accessibility (in all its forms) of such technology should therefore be foregrounded (Emezue et al., 2022; Sabri et al., 2023; Sumra et al., 2023).

### Ethical Issues of Technology

Victim-survivors’ use of digital technologies raises numerous ethical issues. When considering the development of technology such as the chatbot, research suggests that safeguards to ensure that victim-survivors are not subjected to further risk should be implemented (Eisenhut et al., 2020). This is particularly important to mitigate against perpetrators’ use of technology to further abuse victim-survivors (Afrouz, 2023; Rogers et al., 2022), for example, via malicious dual-reporting (reported previously by Brooks-Hay & Kyle, 2015 in nontech reports) and/or use of private data as a tool of control (Woodlock et al., 2020). This is especially important as some victim-survivors do not have private access to their own phone, something which is assumed by most apps (Westmarland et al., 2013). Suggestions for safeguards include hidden areas within apps, password-protection, and the ability for push-notifications to be disabled (Brignone & Edleson, 2019). Keystrokes and contextual cue detection (the particular way or words a person types on a keyboard) to ascertain that it is a victim-survivor accessing the chatbot can also be implemented alongside measures such as automatic data deletion to protect victim-survivors if passwords are continually entered incorrectly, and a quick-exit button if victim-survivors feel unsafe when using technology (Freed et al., 2018).^3^ DVA app developers should also be aware that perpetrators may check victim-survivors’ internet history (Douglas et al., 2019), and should embed information about how to delete this, or ensure that it is not stored in the first place.

Victim-survivors report many ways that they feel unsafe when using technology. In addition to concerns that personal data entered may not be private and secure and/or that they will not be able to delete data off devices, they fear being tracked by perpetrators (Afrouz, 2023; Sabri et al., 2023; Xu et al., 2021). A key feature of many apps is the “location services” function, which serves to provide people with information about their local area, such as DVA support services. This function could, however, be used by perpetrators to track victim-survivors’ movements (Afrouz, 2023; Brignone & Edleson, 2019; Douglas et al., 2019; Draughon-Moret et al., 2022), jeopardizing their safety.

### The Need to Provide a Trauma-Informed Service

Technology developed for use by victim-survivors who have been subjected to DVA must be trauma-informed, that is, working from a victim-survivor-centered perspective, recognizing the harm caused by DVA, and not causing further trauma for victim-survivors by their use of the technology. Within a recent research study by Domingo-Cabarrubias et al. (2023), participants felt that the “technological innovations used in domestic and family violence were not well researched based on the specific needs of victims, and they were not developed in a survivor-led and trauma-informed manner” (p. 5).

Research suggests that some current DVA apps are insensitive and inaccurate (Park & Lee, 2021). When considering language use, it should be empathic, reaffirming, nonjudgmental, sensitive, friendly, and validating (O’Campo et al., 2021; Park & Lee, 2020; Sabri et al., 2023; Tarzia et al., 2017). Ragavan et al. (2020) found during their development of a health education app that being trauma-informed relates not just to language used within the app, but also other types of media (examples could be imagery or video). Platforms should also be able to support multiple conversations without glitches, such as not causing conversations to time out too quickly or disconnecting the user after a short amount of time.

### ISEDA and Developing a Chatbot

This article explores the challenges that women who are seeking help for DVA may experience, examining whether a chatbot can offer positive solutions alongside highlighting our findings relating to some of the concerns victim-survivors have about its creation and implementation.

#### Working Across Multiple Countries: Definitional and Legal Considerations

The ISEDA project involved working across seven European Countries. In a project of this scale, encompassing multiple countries and finding commonalities across governments, legislation, and police practices can be challenging. Even so, we know that all the countries present in the study have a high number of regulations and legislation on DVA (see Vicente-García et al., 2023). The national laws have a broad spectrum of action on DVA, including crime reporting procedures, investigation procedures, and prevention programs. In addition to national-level policies, several of the countries, including Catalonia (Spain), Scotland, and Italy, have a local planning and policy framework on DVA (Vicente-García et al., 2023).

Definitions of violence against women are culturally, historically, and spatially specific (Hester & Westmarland, 2005). Discussions began at the inception of the project to use European-wide definitions (even though one country was no longer in the European Union [EU]) which incorporated DVA as a gendered issue and one which encompassed patterns of abusive behaviors. It is recognized that while all the countries in the EU, and those involved in this study, share similar definitions, different countries have distinct policies and approaches to combat DVA. For the purposes of this article, we use the common definition adopted by the Council of Europe Convention on Preventing and Combating Violence against Women and Domestic Violence (Istanbul Convention) 2011 (Council of Europe, 2011) entered into force in 2014, to understand violence against women in general and DVA in particular:

“violence against women” shall mean a violation of human rights and a form of discrimination against women, and shall mean all acts of gender-based violence that involve or are likely to involve harm or suffering of a physical, sexual, psychological or economic nature to women, including threats of such acts, coercion or arbitrary deprivation of liberty, in public or private life; and coercion or arbitrary deprivation of liberty, in public or private life;

“domestic violence” shall mean all acts of physical, sexual, psychological or economic violence occurring within the family or household or between former or current spouses or domestic partners, regardless of whether the perpetrator shares or has shared the same domicile as the victim.

(Council of Europe Treaty Series - No. 210 https://rm.coe.int/168008482e)

Whilst the ISEDA chatbot is based on artificial intelligence (AI), it is not an AI Chatbot. It uses AI for intent and entity detection only and not for answer generation; the answers have been preprogrammed by the ISEDA team. The purpose of the chatbot is to enable victim-survivors to search for information in relation to DVA, such as local legislation, victim-survivors’ rights, and information about local support services. The chatbot is initially being piloted in Bulgaria, Catalonia, and Greece, before being rolled out (if successful) to other European countries in the future. It aims to be a particularly supportive tool for women in countries where provision and services are less widespread due to lack of funding. We will now discuss the methodology used within the study, before moving on to presenting the findings.

## Methodology

Interviewers from seven participating countries conducted either focus groups and/or 1:1 interviews^4^ with a total of 65 victim-survivors (see Table 1) aged between 22 and 69 years. Interviews with police authorities and civil service/non-governmental organizations were also conducted but do not form part of this article. All victim-survivors were provided with a comprehensive information sheet and consent form. Written or oral consent was received prior to participation so interviewees knew what participation would involve. They were also given a list of main themes so they could prepare beforehand if they wished—each country followed the same interview schedule. Before the interview, all participants were told that they could omit any questions that they preferred not to answer. If they wished, participants could take a break or end the interview at any time. Relevant to this article, interviewers in each country asked victim-survivors a question along the lines of “What is your opinion on the use of apps, chatbots, and other digital communication tools during the reporting process?” Immediately after the interview ended, the interviewer asked participants how they were feeling to ensure they felt emotionally stable enough to end the interview. Following the interview, all participants were provided with information of local support organization information to access if they wished.

**Table 1.** Methods Used by Each Country.

Country	Interviews with victim-survivors	Focus groups with victim-survivors	Total number of victim-survivors
Bulgaria	0	3 (involving 20 participants)	20
Italy	6	0	6
Finland	2	1 (involving 3 participants)	5
Greece	0	2 (invoving 9 participants)	9
Scotland	6	0	6
Cyprus	0	1 (involving 6 participants)	6
Catalonia	0	2 (involving13 participants)	13

Interviewers from each country then transcribed interviews and used descriptive thematic analysis to theme them. Codes were generated iteratively based on input from the questions in the interview scripts and emergent themes. Some of the codes were structural (based on the questions asked), and others were more analytical (arising from the emergent themes). This resulted in the creation of an analysis handbook that was applicable across the seven countries, yet was also context specific, allowing for comparison of themes. Interviews which were not conducted in English were translated. Themes were then provided to academic researchers in Catalonia who created a global report combining the key findings.

## Findings

During the interviews and focus groups, victim-survivors discussed their thoughts on the use of chatbots as a tool for reporting DVA to the police, for finding information about DVA and storing evidence that could potentially be used in court. They discussed both their understandings of the positives and negatives of these types of tools which are relayed below.

### Positive Perceptions of Chatbot Technology

#### Chatbots Offer Anonymity and Nonjudgment

Victim-survivors felt that the use of a chatbot to “talk to” would be positive in the sense that it would allow them a sense of anonymity, which they said would increase their confidence to be open about the abuse they were experiencing. Alongside this, they thought a chatbot would be less judgmental than a real person:

I lacked the courage to talk about it with someone, it's one thing over chat as you don't see the person and you don't know them, it's simpler. On the other hand, talking about it with an acquaintance / a person face-to-face is much more difficult, so I think it would have helped me. (Italy, ID1)

You call the family counselling clinic and then they say, ‘Nonsense, try to get along’, or to the deaconess of the church and they say, ‘Nonsense, try to get along’. So, in a way, the bot would never respond like that. It would try to solve the situation. (Finland, ID10)

The universal experience of victim-blaming was shared by women across Europe. In designing an app to support women, we need to be cognizant of what prevents them from seeking help. Being asked why it has taken them so long to act, or questioning their behaviors in relation to the abuse they have been subjected to inhibits women from calling for help, especially when children are involved (see Hester, 2011; Katz, 2015; Morrison et al., 2013; Mullender, 1996).

#### Chatbots as Providing an Immediate and Accessible Response

Victim-survivors commented on the accessibility of chatbots, in relation to being able to use them at a time that was convenient for them. This was particularly important for women who still lived with controlling, abusive partners who may be confining women to the house. One woman felt that reporting an abusive perpetrator via a chatbot (rather than speaking on the phone) meant that the perpetrator would be more likely to be at home when the police arrived, which she saw as a positive, whereas others felt that reporting via the chatbot meant that the perpetrator would not know they had reported:

Well, the fact that on the other side there is a computer can help a person who cannot move from home because of their partner's control, in such cases it can be very helpful. Even for me it was difficult to leave home to go to the police, it probably would have made it easier for me [to have a chatbot]. (Italy, ID6)

It would be good to have the chance to inform electronically the police, in order the perpetrator to be at home when they come. Otherwise, he knows that I called the police. (Greece, ID2)

I was afraid to go out of my home. The abuser was very scary. He threatened me by knifes. I was not able to submit a report because I was afraid. I was not able to go out of my home. (Bulgaria, ID5)

Victim-survivors should feel listened to and reassured when using technology (O’Campo et al., 2021). In this way, a chatbot, as opposed to a phone call could give women the opportunity to feel connected and responded to. The quotes above demonstrate how the immediacy of action is significant. However, it is key to note that women do not just want to input data into a tool, rather they want a qualified response to their call for help and / or relevant and helpful information to be provided.

#### Chatbots as Providing a Central Recording System for Criminal Processes

Research documents how women are required to repeat their stories of abuse at various stages of the criminal justice process (Forbes, 2021; Lombard et al., 2023). Participants within this research welcomed the idea of all of the information related to their case being stored in one place, so both they and the agencies associated with their case could access and update it. This was seen as convenient, rather than women having to sit, for example, in a phone queue waiting to speak to someone. For these reasons, having a central recording system could reduce trauma for women:

The bot can be utilized in a way that all contacts are recorded. So, if the authorities have access to the bot's data, so to speak, they can easily get those contacts as evidence, and they will still be in writing. (Finland, ID8)

Some cases can take years from report to finalization (even more so since COVID-19), meaning women bear huge emotional burdens associated with retelling as well as facing anxiety waiting for updates from various professionals involved in their case, described as legal systems abuse (Douglas, 2018) and “paper abuse” (Miller & Smolter, 2011). Uploading information to the chatbot was also perceived as a way to reduce the emotional strain on victim-survivors due to only needing to, for example, provide information once, and act as a central location to record important information such as photos, videos and other information to serve as evidence of the abuse (see Douglas & Burdon, 2018 and Douglas et al., 2019). Lombard and Proctor (2024) discuss the burden of “justice work” upon victim-survivors and this is one way that it could be mitigated.^5^ However, it is crucial to understand that different countries have different legal jurisdictions and not all are able to legally use the chatbot data as evidence of a crime or within legal proceedings.

#### “‘Better than Nothing”’

When discussing the use of chatbots, women judged them as a useful educative tool to access information about DVA. The majority of women spoken to highlighted a preference for the chatbot to be accessed for information rather than using it to report a crime. It is crucial to understand not all women want to report their abuse, either because of previous bad experiences with the justice systems or because they do not want to criminalize their partner / ex-partner (see Lombard & Proctor, 2024). For some women the chatbot offered the potential for first step support rather than as a catch all solution:

For getting help at early stage … I consider AI [Artificial Intelligence] at an early stage where a person or a woman realizes that everything is not okay … there would be more information available, and it would not be so taboo about these things that women would understand what is right or OK and what is not OK … and that artificial intelligence will then take you further down that path and then possibly tell you which parties you could get help with this matter. (Finland, ID10)

Women in one country who felt that existing DVA support was poor felt that a chatbot would be ‘better than nothing’:

Just to remind you all that only [a] few of us received humane support. … so instead of nothing, it is better to have at least something that I can be provided with guidance. (Cyprus, ID1)

Two participants in Scotland mentioned that tools like chatbots would be most useful to support younger women who are more likely to use technology, in line with suggestions from existing research (Park & Lee, 2021; Storer et al., 2022; Tarzia et al., 2017):

Some people, young people, for example do everything through IT [Information Technology] and apps or whatever. I see them at the school, they pay [for] everything with their phones. (Scotland, ID5)

So, I think we can use our technology to support our younger generations and our older generations to understand more about what is coercive control, how does that look and feel … just to support people and women and girls and obviously other people who are in domestic abusive relationships to understand what it is and how can you report. (Scotland, ID6)

Although the participants covered a wide age range, they shared an awareness of the intersectional disparities around phone use, access to technology and awareness of apps and how to use them. All are relevant when understanding how, when and in what ways women would access the chatbot and make best use of its capabilities.

### Concerns About Chatbot Technology

In addition to the potential benefits of chatbots, women discussed pitfalls. These primarily revolved around issues of data security, concerns with the perpetrator accessing the chatbot and whether or not a chatbot can mimic human empathy. There were differences in the opinions of victim-survivors across Europe. For those who lived in countries with definitive DVA laws and legislation and funded public services, they were more likely to see the chatbot as “complementing” existing support mechanisms. For those that did not have workable and accessible services, chatbots were seen as a solution where no other option existed.

#### Cannot Replace Support from a Human

Some victim-survivors felt unsure about chatbots and stated that they would prefer to speak to a human rather than receive help through a “robot.” In particular, it was argued a chatbot could not understand complexity and emotion relating to their situation that can be picked up when speaking to a human, especially when discussing DVA:

For this kind of thing, I think it's better face to face, because perhaps it's better to make the emotions felt and the gravity of the situations better understood, it gives me the idea that face to face is better. I don't know if a chatbot could be more helpful, I'm dubious about it. (Italy, ID3)

People can show emotion better [in-person], which is what you need, you do not need kind of faceless, you need people that when you are saying something to them they try very hard to look professional while clearly being horrified, which is quite validating and makes it easier to keep talking … you manage to say more than if you were writing it down. (Scotland, ID1)

I’m an advocate for improved technology but sometimes when you are going through a horrendous abusive relationship, a bit of human kindness and support is what you are looking for and it's what you need to then take you to the step of reporting and then for the treatment, again, improvement in consistency. (Scotland, ID5)

An important characteristic is the emotional intelligence that I don’t know if a robot can do, since even people fail to have. (Catalonia, ID1)

In addition to concerns about chatbots being unable to replicate human empathy, some women also worried chatbots may misinterpret the information they were receiving. This may present itself in terms of material that was input being minimized, meaning that women may not be directed to relevant in-person support. This theme was particularly prevalent for Finland and Scotland:

There is a risk that the bot will misinterpret the issue. There's the risk of misinterpretation. Some word or some connection, and then the victim-survivor does not receive the kind of help she needs. (Finland, ID8)

I don’t think a robot could do that job, it really, there's no many nuances to people, to people basically, people in trouble, tiny little whispers, scratches, vulnerable callers, no there's too many variables when it comes to crime and people in distress and victims. (Scotland, ID7)

The level of tailored support available to women in both Finland and Scotland goes some way to understanding why those interviewed within these countries did not want to rely solely upon technology to provide and facilitate support.^6^

#### Safety and Security

Victim-survivors discussed security considerations relating to using a chatbot. Some women were concerned whether personal information entered into the chatbot would be safe or could be hacked into by perpetrators, whereas others worried their mobile devices may be taken away or monitored:

They always take the phone from the hand. It's the way of reacting when the violence has started, and you've said you will call somewhere. The first thing they do is take the phone and break it down. (Finland, ID5)

There's so much scamming and frauding and you know, I’m actually, I no longer use my phone. I no longer use online banking, just because of the amount of scamming and frauding and what's to say that a hacker can’t hack into the AI and you know that could go horribly wrong. (Scotland, ID7)

There is a risk that a wrong person gains access to someone else's mobile device - technology rarely is completely flawless, there are always risks. (Finland, ID8)

When discussing inputting information into the chatbot, some respondents were concerned that sending information digitally means waiting for a call back or reply which would be anxiety-inducing and leave the victim-survivor in a state of limbo, compared to the immediate feedback of reporting to a person:

Waiting for an online reply or a call back would have had me unable to function, honestly at that time. I would have felt in a state of fear in absolute limbo. I wouldn’t have been able to move away from whatever technology I did have at that time. (Scotland, ID5)

## Conclusions: Moving Forward With Technological Advances and the Development of a Chatbot

The findings from this paper have implications for the development of the ISEDA chatbot and also more broadly in terms of technological innovations to support victim-survivors who are subjected to DVA. We know from previous research and our own data that technology (such as chatbots) can be useful to support people subjected to DVA, by being easier to open up to than speaking to a human, being more convenient and by feeling more private and less judgmental. Our interviews with victim-survivors detailed their priorities relating to accessibility and ensuring that tools are empathic, relevant, and kept up-to-date. The women we spoke to also felt innovations in technology could enable them to access the police more safely than ‘traditional’ mechanisms such as a telephone call. However, it is also important to note, from research relating to women's interactions with criminal justice systems, that feedback or constructive interaction with a person is not always immediate or forthcoming in real life (Lombard et al., 2023), and thus, this concern may not be limited to digital technology only. All were insistent however that technology should not be a replacement for human contact.

Following feedback from victim-survivors and wider public agencies, the purpose of the ISEDA chatbot was refined from being ‘responsive’ to be more informative. Our current aim in light of such responses is for it to enable victim-survivors to search for information in relation to DVA, such as local legislation, victim-survivors’ rights and information about local support services. We anticipate that the tool will be particularly useful for victim-survivors in countries where, primarily due to lack of funding, there are little to no existing support mechanisms. Further research could test this theory in more countries to assess the generalizability of the finding as well as to suggest any country-specific considerations that should be considered when developing technology-based supportive tools for DVA.

A priority of the ISEDA project was that any solutions created to support help-seeking should prioritize the safety of victim-survivors. In keeping with this precedent and alongside trauma-informed practice, the responses of the victim-survivors in this research fed directly into the design and implementation of the solution meaning that their worries and concerns were addressed rather than overlooked. Technology developers in the area of DVA generally should be cognizant of malicious use of such data tools by perpetrators, and ensure that in-built safeguarding is implemented. As part of this, it should be clear to victim-survivors who use any form of technology how their data is stored and used, and clear instructions for how they can utilize safety mechanisms which are built into the platform. Furthermore, careful consideration should be given to platform hosts, ensuring organizations are ethical and place victim-survivor safety and security as paramount. When considering hosting options, for any technological solution, it is vital that the platform and data ownership decisions are communicated to victim-survivors, as many (illustrated within this and other research) have concerns about ensuring that their data is private and secure when using technology (Sumra et al., 2023). Existing research already makes suggestions as to how this can be addressed, such as passwords, disabling of push-notifications, a quick-exit button and keystroke contextual cue recognition (Brignone & Edleson, 2019; Freed et al., 2018) but those working in technology, with little or no knowledge of DVA need to be particularly mindful.

Chosen solutions to addressing DVA whether online or in real life should place women's safety at the center. Furthermore, when developing new technology to respond to DVA, the preparedness of police resources to manage potentially increased reports from victim-survivors must be considered. This can be linked to wider issues regarding the continuation and updating of live technological tools. Women's specialist domestic abuse services possess up-to-date, trauma-informed knowledge about gender-based violence and abuse, as well as working closely with victim-survivors, and thus in one respect are well-placed to integrate and manage such tools. However, most are already working at or beyond capacity and are under-funded, and thus integrating the chatbot would place strain on their organizations, as well as requiring high levels of technical expertise (which may or may not already be held by professionals in the organizations). Future research could explore the potential for women's services to coordinate and manage new technological innovations alongside their existing services, focusing on what factors are needed to ensure success.

In terms of our research, the chatbot has the potential to be an innovative solution but also the prospect to act as a stop gap for systems unwilling to invest in transformative measures to eliminate DVA. We need to ensure the chatbot and further technological solutions in this area succeed in helping the women as they are intended to, but that it is not a short-term, underfunded solution that fails to provide trauma-informed support for victim-survivors. For this reason, we argue that specialist women's DVA services should be fully funded to enable them to recruit staff to manage technological tools effectively to support their ongoing work. They should also be funded to be trained and/or to be able to work collaboratively with technology companies to ensure such technological tools remain fit-for-purpose. Funding should not, however, be taken away from other vital work that these services are currently providing, but instead should be in addition.

## Notes

1. See for example *rAInbow: Chatbot To Support Victims Of Domestic Abuse*, s. f.

2. The public story being one of DVA being a problem for cis heterosexual, white, able-bodied, women, from cis heterosexual white, able-bodied men.

3. The ISEDA chatbot is a website, without any password. The website creates a new conversation every time the site is opened. The users are asked not to send any personal data to the chatbot. ISEDA chatbot has a quick-exit button and the chatbot can also be closed and started again while no history remains. Thereby the chatbot has in-built safeguarding and clear instructions for how to use the safety mechanisms which are built in the system (see Brignone & Edleson, 2019; Douglas et al., 2019; Freed et al., 2018). The ISEDA chatbot is programmed to identify the intentions of the users and currently it can answer most of the questions that it is trained to answer. All answers are written in the system. The next step is to widen the collection of potential questions and the selection of answers. The aim is to be able to answer all possible questions to meet country specific scenarios.

4. This came down to victim-survivor preference, availability of researchers and the choices of the research teams.

5. The ISEDA chatbot does not save evidence. The evidence may be downloaded when reporting to police by filling out a form which is in the “Make a report” page of the main web page.

6. In Finland, the public basic services, including social and health care, the police and psychiatric services are at the front line to encounter victim-survivors. These services are in a key position to direct victim-survivors to special services, including shelters and multiagency support. Shelters (crisis work) and outpatient services are organizations that specialize in violence work and provide free crisis assistance and services for those who have experienced violence or the threat of violence in family and close relationships. In Scotland, third-sector organizations support women to make their own choices. They work with women to remain in their homes, find refuge and report their abuse (if they choose to). The Domestic Abuse Scotland Act provides legislation that can be used to prosecute men for their abuse, should women report and if the case makes it to court.
